# Discrimination and Risk of Incident Disability in Older African Americans

**DOI:** 10.1093/geroni/igab046.998

**Published:** 2021-12-17

**Authors:** Brittney Lange-Maia, Melissa Lamar, Sue Leurgans, Aron Buchman, Lisa Barnes

**Affiliations:** 1 Rush University Medical Center, Chicago, Illinois, United States; 2 Rush Alzheimer's Disease Center, Rush University Medical Center, Rush Alzheimer's Disease Center, Illinois, United States; 3 Rush University, Chicago, Illinois, United States

## Abstract

Discrimination is linked to poor health outcomes, but most studies examine young or midlife populations. We assessed associations between discrimination and disability in African Americans. The Detroit Areas Study Everyday Discrimination Scale quantified experiences of interpersonal mistreatment. Separate Cox-proportional hazards models tested the associations between baseline discrimination and incident mobility, activities of daily living (ADLs), and instrumental activities of daily living (IADLs) disability, adjusting for age, sex, education, BMI, smoking, depressive symptoms, and vascular diseases. At baseline, 441, 674, and 469, participants were initially free of mobility, ADL, and IADL disability, respectively, and 257, 185, and 269 new cases of mobility, ADL, and IADL disability were observed over approximately 8.5 years. Discrimination was associated with higher risk of ADL disability (hazard ratio: 1.03 per 1-point higher discrimination score, 95% confidence interval: 1.00-1.06) but no other disability type. Everyday discrimination is associated with risk of ADL disability.

